# Empirical Modeling of the Drying Kinetics of Red Beetroot (*Beta vulgaris* L.; Chenopodiaceae) with Peel, and Flour Stability in Laminated and Plastic Flexible Packaging

**DOI:** 10.3390/foods13172784

**Published:** 2024-09-01

**Authors:** Elisabete Piancó de Sousa, Emanuel Neto Alves de Oliveira, Thamirys Lorranne Santos Lima, Rafael Fernandes Almeida, Jefferson Henrique Tiago Barros, Clara Mariana Gonçalves Lima, Angelo Maria Giuffrè, Jolanta Wawrzyniak, Sławomir Wybraniec, Henrique Douglas Melo Coutinho, Bruno Fonsêca Feitosa

**Affiliations:** 1 Federal Institute of Education, Science and Technology of Rio Grande do Norte, Pau dos Ferros 59900-000, RN, Brazil; elisabete.pianco@ifrn.edu.br (E.P.d.S.); emanuel.oliveira16@gmail.com (E.N.A.d.O.); 2 State University of Paraíba, Lagoa Seca 58117-000, PB, Brazil; thamirysl2012@hotmail.com; 3 Department of Food Engineering and Technology, Faculty of Food Engineering, University of Campinas, Campinas 13056-405, SP, Brazil; rafaelfernandes.creajrba@gmail.com; 4 Federal Institute of Education, Science and Technology of Acre, Rio Branco 69918-064, AC, Brazil; jefferson.barros@ifac.edu.br; 5 Department of Biological Chemistry, Cariri Regional University, Crato 63105-000, CE, Brazil; clarmarianalima@gmail.com (C.M.G.L.); hdmcoutinho@gmail.com (H.D.M.C.); 6 Department of Agraria, University of Studies “Mediterranea” of Reggio Calabria, 89124 Reggio Calabria, Italy; 7 Faculty of Food Science and Nutrition, Poznań University of Life Sciences, 60-624 Poznań, Poland; jolanta.wawrzyniak@up.poznan.pl; 8 Department of Chemical Technology and Environmental Analysis, Faculty of Chemical Engineering and Technology, Cracow University of Technology, Warszawska 24, 31-155 Krakow, Poland; slawomir.wybraniec@pk.edu.pl; 9 Department of Agricultural Engineering, State University of Amapá, Amapá 68950-000, AP, Brazil

**Keywords:** storage, food waste, drying kinetics, physicochemical properties, agro-industrial waste

## Abstract

Despite the high global production of beetroot (*Beta vulgaris* L.), its peel is often discarded. Transforming beetroot into flour can reduce waste, improve food security, and decrease environmental pollution. However, large-scale feasibility depends on understanding drying kinetics and optimal storage conditions. This study aimed to investigate the effects of different temperatures in the convective drying of whole beetroot and evaluate the influence of laminated flexible and plastic packaging on flour stability over two months. Drying kinetics were analyzed using five models, with the Page and Logarithm models showing the best fit (*R*^2^ > 0.99). *D_ef_* values (1.27 × 10^−9^ to 2.04 × 10^−9^ m^2^ s^−1^) increased with rising temperatures while drying time was reduced (from 820 to 400 min), indicating efficient diffusion. The activation energy was 29.34 KJ mol^−1^, comparable to other plant matrices. Drying reduced moisture and increased ash concentration in the flour. The flour showed a good water adsorption capacity and low cohesiveness, making it marketable. Laminated packaging was more effective in controlling physicochemical parameters, reducing hygroscopicity, and maintaining quality over 60 days. In summary, the Page model can predict beetroot drying kinetics effectively, and laminated packaging can control flour stability.

## 1. Introduction

Beetroot (*Beta vulgaris* L.; Chenopodiaceae) is a vegetable primarily composed of water, carbohydrates, vitamins, and minerals. This vegetable has three biotypes with significant economic importance: sugar beets, fodder beets, and horticultural beets [1,2]. Sugar beets are used as an industrial raw material for sugar production. In addition to being more widespread in most countries, their production is estimated at 280 million tons per year [3,4]. Brazil primarily produces the horticultural biotype (red beetroot), especially the cultivar Early Wonder [5]. The beetroot cultivation area in Brazil is approximately 18 thousand hectares, with each hectare annually yielding about 30 tons of this vegetable [6].

Despite the high production of beetroot, its peel is an agro-industrial by-product that is often discarded, generating economic impacts [7]. Beetroot peel is a source of fibers, calories, and natural pigments such as betalains, which have antioxidant properties and help prevent cardiovascular and neurodegenerative diseases [8,9,10]. Therefore, the complete utilization of beetroot in the form of food flour can be a viable alternative to food waste, food insecurity, and environmental pollution [11,12,13,14].

Flour is an industrial product that requires low-cost production and has high market acceptance [15,16,17,18]. However, the feasibility of integrating whole beetroot into this large-scale process depends on understanding its drying kinetics, including the application of empirical mathematical models and other factors such as effective diffusion coefficient (*D_ef_*) and activation energy (*Ea*) [19,20,21]. Currently, the beetroot flour market is still a limited, costly, and poorly consolidated segment. This alternative product offers health benefits to consumers, which are associated with its antioxidants, fibers, and minerals, such as iron and potassium [22,23].

Techno-scientific research has focused on the application of sugar beet flour in gluten-free products such as baking doughs [8,22,23,24,25,26,27]. Meanwhile, other studies are limited to obtaining the flour and evaluating its physicochemical parameters [1,9,10,13,28]. It is worth noting that the increase in veganism and vegetarianism can also boost the demand for beetroot flour as a natural ingredient in plant-based and meat-alternative products. It is important to note that there are no studies in the literature that have evaluated the drying kinetics of whole beetroot nor the storage stability of the resulting flour under different packaging conditions.

Therefore, this study aimed to evaluate the effects of different temperatures on the convective drying of whole red beetroot, as well as determine the drying kinetics and analyze it in terms of mathematical modeling, *D_ef_*, and *E_a_*. This study also aimed to evaluate, for the first time, the influence of packaging on the stability of the flour over 60 days of storage.

## 2. Materials and Methods

### 2.1. Materials

The raw material used was horticultural beetroot (*B. vulgaris* L.), selected without mechanical damage and at full physiological maturation. In other words, complete physiological ripeness was determined based on several factors: the uniform deep red color of the root, the firmness of the flesh, and the size of the beetroot, which typically ranged between 5 and 10 cm in diameter. These criteria are indicators that the beetroot has reached full maturity, ensuring optimal pigment concentration and nutrient content. It was acquired from three distinct batches at a farmers’ market in the city of Pau dos Ferros, Rio Grande do Norte, Brazil (Latitude: 6°6′9″ S, Longitude: 38°12′33″ W).

### 2.2. Preparation and Characterization of Whole Beetroot Paste (WBP)

The beetroots were subjected to washing under running water, sanitization by immersion (in a 50 ppm sodium hypochlorite solution for 15 min), rinsing under running water, and grinding in an industrial blender LS6 (Skymsen^®^, Brusque, Brazil) to obtain a homogeneous paste. It is worth noting that the grinding was performed on whole beets, including the peel. This WBP was characterized, in three repetitions, for its physicochemical parameters: water content (drying in an oven at 105 °C for 24 h); ash content (incineration in a muffle furnace at 550 °C for 6.5 h); pH (digital pH meter, previously calibrated with buffer solutions of pH 4.0 and 7.0); Titratable Total Acidity (TTA) in % citric acid, through titration with standardized 0.1 N NaOH; and total soluble solids (TSSs), by refractometry [29]. Water activity (a_w_) was analyzed using a portable water activity analyzer LabStart-a_w_ at 30 °C (Novasina^®^, Lachen, Switzerland).

### 2.3. Drying Kinetics

The drying of WBP was carried out in an air circulation oven (TE-394/3MP, TECNAL^®^, Piracicaba, Brazil) at temperatures of 60, 70, and 80 °C, according to Almeida et al. [30] and Thao et al. [31]. The air velocity was 1.0 m s^−1^. The paste was spread evenly on rectangular stainless-steel trays (24.5 × 16.5 cm), forming a thin layer with a thickness of 0.5 cm. During drying, the trays were weighed at regular time intervals until the mass readings became constant.

### 2.4. Mathematical Modeling

From the experimental drying data, the moisture ratio (MR) values were calculated according to Equation (1) [29]. The empirical mathematical models of Page [32] (Equation (2)), Midilli [33] (Equation (3)), Lewis [34] (Equation (4)), Two terms [35] (Equation (5)), and Logarithm [36] (Equation (6)) were calculated using the Quasi-Newton method. The coefficient of determination (*R*^2^) (Equation (7)), Mean Square Deviation (MSD) (Equation (8)), and reduced chi-square (χ^2^) (Equation (9)) were adopted as criteria to evaluate the fit of the models to the experimental data.

(1) $$MR=\frac{\left(X_{t}-X_{e}\right)}{{(X}_{0}-X_{e})}$$

(2) $$MR=a\times exp\left(-kt^{n}\right)$$

(3) $$MR=a\times exp\left(-kt^{n}\right)+b\times t$$

(4) MR=exp(−kt)

(5) $$MR=a\times exp(k_{0}t)+b\times exp(-k_{1}t)$$

(6) $$MR=a\times exp\left(-kt\right)+c$$

(7) $$R^{2}=\frac{{\sum_{i=1}^{N}\left[{(MR}_{exp}-\overset{¯}{{MR}_{pre}})({MR}_{pre}-\overset{¯}{{MR}_{pre}})\right]}^{2}}{{\sum_{i=1}^{N}\left({MR}_{exp}-\overset{¯}{{MR}_{pre}}\right)}^{2}{\sum_{i=1}^{N}\left({MR}_{pre}-\overset{¯}{{MR}_{exp}}\right)}^{2}}$$

(8) $$MSD=\sqrt{\frac{\sum {\left({MR}_{pre}-{MR}_{exp}\right)}^{2}}{N}}$$

(9) $$X^{2}=\frac{1}{N-n}\sum_{i=1}^{N}{\left({MR}_{pre}-{MR}_{exp}\right)}^{2}$$

where (1) *Xt*, *Xe*, and *X*_0_ are the moisture content at time *t*, the equilibrium moisture content, and the moisture content in *t* = 0, respectively; (2)–(6) *t* is the drying time (min); *k* is the drying constant (min^−1^); *n*, *b*, *c*, and *a* are constant in the models; (7)–(9) *MR_exp_* and $\bar{{MR}_{exp}}$ are the ratios of the experimental moisture content in the times and its average, respectively; *MR_pre_* and $\bar{{MR}_{pre}}$ are the ratios of the moisture content predicted by the equation in the times and its average, respectively; *N* is the number of observations made during the experiment; and *n* is the constant number in the model.

### 2.5. Effective Diffusion Coefficient (D_ef_) and Activation Energy (E_a_)

The data were fitted to Fick’s diffusion model, considering the flat plate geometric shape with a five-term approximation, as shown in Equations (10) and (11).

(10) $$\ln\left(\frac{X}{X_{0}}\right)=\ln\left(\frac{8}{\pi^{2}}\right)-\frac{\pi^{2}}{{4L}^{2}}D_{ef}\times t$$

(11) $$\alpha =-\frac{\pi^{2}}{{4L}^{2}}{\times D}_{ef}$$

where *D_ef_* is the effective diffusion coefficient; *L* is the characteristic length (half the thickness of the sample); and α is the slope of the linear fit of the $\ln\left(\frac{X}{X_{0}}\right)\;$ data as a function of time.

In relation to the dependence of *D_ef_* on temperature, the expression described by Arrhenius was applied (Equation (12)).

(12) $$D_{ef}=D_{0}\times exp\left(-\frac{E_{a}}{R(T+273.5)}\right)$$

where *D*_0_ is the pre-exponential factor and *R* is the universal ideal gas constant (8.314 J mol K^−1^).

The *E_a_* was calculated from the linearization of the equation’s coefficients, applying the logarithm according to Equation (13) [37].

(13) $$ln{\;(D}_{ef})={lnD}_{0}-\frac{E_{a}}{R}\times \frac{1}{\left(T+273.15\right)}$$

By plotting *ln* (*D_ef_*) as a function of the inverse of the absolute temperature (*T*), the slope of the linear regression provides −*E_a_*/*R*.

### 2.6. Processing and Quality Characterization of Whole Beetroot Flours

The samples dried at 60 °C were removed from the trays using a stainless-steel spatula and ground in a food processor (R12134, Philips^®^, Amsterdam, The Netherlands) to obtain the food flour. The quality of the flour was evaluated in three repetitions according to the Association of Official Analytical Chemists [29]. The physicochemical parameters evaluated were water content, total solids, ash, pH, TTA, TSS, and a_w_, as described in Section 2.2. Additionally, the physical parameters of bulk density (ρB), tapped density (ρC) [38], Carr index (CIn) [39], Hausner ratio (*HR*) [40], wettability (*W*) (adapted from Freudig et al. [41]), and solubility (*S*) [42,43] were evaluated, respectively, according to Equations (14)−(19). Insolubility (*I*) was determined based on the percentage of material not solubilized after one minute of stirring [44].

(14) $$\rho B=\frac{w}{V_{t}}$$

(15) $$\rho C=\frac{w}{V_{c}}$$

(16) $$CI\left(\%\right)=\left(\frac{\rho C-\rho B}{\rho B}\right)\times 100$$

(17) $$\mathrm{HR}=\frac{\rho C}{\rho B}$$

(18) $$W=\frac{w}{t}$$

(19) $$S=\left[\left(\frac{w_{ds}}{w}\right)\times 4\right]\times 100$$

where *w* is the sample weight (g); *V*_t_ is the total volume (cm^3^); *V*_c_ is the occupied volume (cm^3^); *t* is the time (s); and *w*_ds_ is the weight of dissolved solids in the supernatant (g).

### 2.7. Stability of Flours in Laminated and Plastic Flexible Packaging

The packaging of 20 g of food flour was carried out in laminated and plastic flexible packaging, equipped with a zip lock and measuring 12 cm in width and 17.5 cm in height, with a capacity of 100 g. The storage took place at room temperature (average of 25 ± 2 °C) for 60 days. Stability analyses were performed in at least three repetitions at times 0 and 1 (initial), and every 30 days. The evaluated parameters were water content, pH, TSS (Association of Official Analytical Chemists, 2016), ρ*B*, ρ*C*, CIn, HR, and W (adapted from Freudig et al. [41] and Achor et al. [45]), as described in Section 2.6.

### 2.8. Statistical Treatment

The empirical modeling of drying kinetics was conducted using the software Statistica 7.0 (StatSoft South America, Porto Alegre, Brazil). The stability results of the flours were analyzed using the software Assistant version 7.7 beta [46], through Analysis of Variance (ANOVA). A Completely Randomized Design (CRD) experiment with three repetitions was conducted in a 3 × 2 factorial scheme, with the factors studied being drying temperature (60, 70, and 80 °C) and different packaging (laminated and plastic flexible packaging). The means were compared by Tukey’s test (*p* < 0.05).

## 3. Results and Discussion

### 3.1. Empirical Modeling of Drying Kinetics

The drying kinetics were studied using five drying models. The constants and statistical parameters of the drying kinetics (60, 70, and 80 °C) of WBP are presented in Table 1. In terms of engineering processes, an *R*^2^ > 0.93 can be considered satisfactory [47]. Therefore, all the tested models showed acceptable *R*^2^ values for predicting the drying kinetic behavior of WBP. However, the Page and Logarithm models stood out among the others, as they achieved *R*^2^ values above 0.99 at all applied temperatures, with the highest *R*^2^ at 60 and 70 °C, respectively. The Midilli model performed better at 80 °C, with an *R*^2^ close to the ideal. The two-term and Lewis models did not indicate good fits for the drying kinetics of WBP compared to the other tested models.

Although it is common to find studies that determine the best predictive model based only on the *R*^2^ value, this practice is not the most appropriate as it is subject to misinterpretations of the fits [20]. Therefore, this study also considered the MSD and χ^2^ values. For MSD, performance was similarly and inversely proportional to *R*^2^, with satisfactory values for all tested temperatures in the Page and Logarithm models (MSD below 0.05). According to Panchariya et al. [48], the best predictive model is the one that delivers the lowest χ^2^ values. In this regard, the Midilli model presented the lowest χ^2^ values at 70 and 80 °C. In turn, the Page model performed better at 60 °C, with satisfactory values for all temperatures (Table 1). The Logarithm model also achieved good fits at 60 and 70 °C.

The drying behavior of the beetroot cubes was well described by the two-term exponential model indicated by a lower mean relative error (7–8%) [21]. It was observed that the drying constant k tended to increase with rising temperature. The value of k depends on the type of product, the temperature, and the relative humidity of the air [49]. This trend is also related to *D_ef_* in the drying process during the falling-rate period and to the net diffusion controlling the process. There was also a reduction in the constant *n* at the intermediate temperature (70 °C), followed by an increase at 80 °C. Perez et al. [50] stated that the parameter *n* is related to the internal resistance of the material to drying.

**Figure 1:**
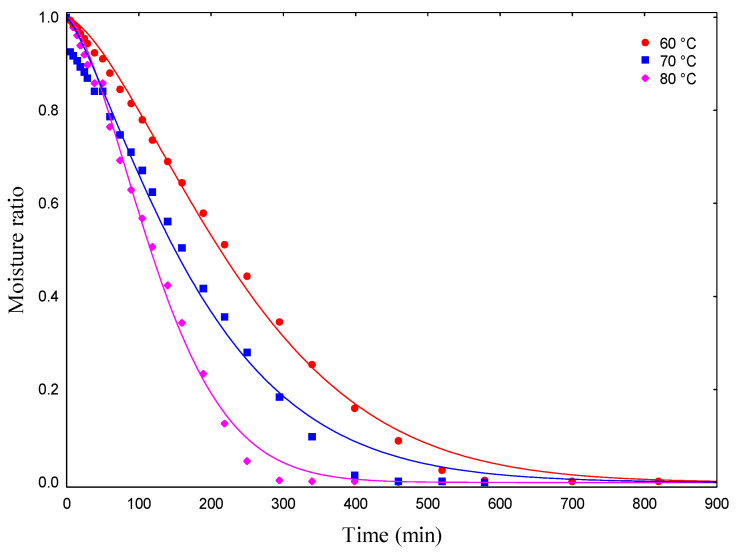
Drying curves (60, 70, and 80 °C) of WBP fitted to the empirical Page model. WBP, whole beetroot paste.

Figure 1 shows the drying curves (60, 70, and 80 °C) of WBP with the fits of the Page model. The curves conform to the typical constant rate drying curve described by Geankoplis [51].

As expected, water losses were greater at the beginning of the drying process. Higher drying temperatures resulted in a faster drying process, which is associated with higher water vapor pressure on the surface [52]. The stabilization phase of drying was slower for WBP dried at 60 and 70 °C. The water removal rate decreased due to the low water content and the difficulty of migrating to the surface of the material. Almeida et al. [20] emphasized that surface water is removed more easily and quickly. Therefore, the drying time can vary according to the material, air velocity, and layer thickness. In this study, the drying time of WBP was 820, 520, and 400 min for temperatures of 60, 70, and 80 °C, respectively. The influence of temperature on reducing drying time is a consequence of the increased vibration level of water molecules, thus contributing to faster water diffusion [53].

### 3.2. Effective Diffusion Coefficient (D_ef_) and Activation Energy (Ea)

**Figure 2:**
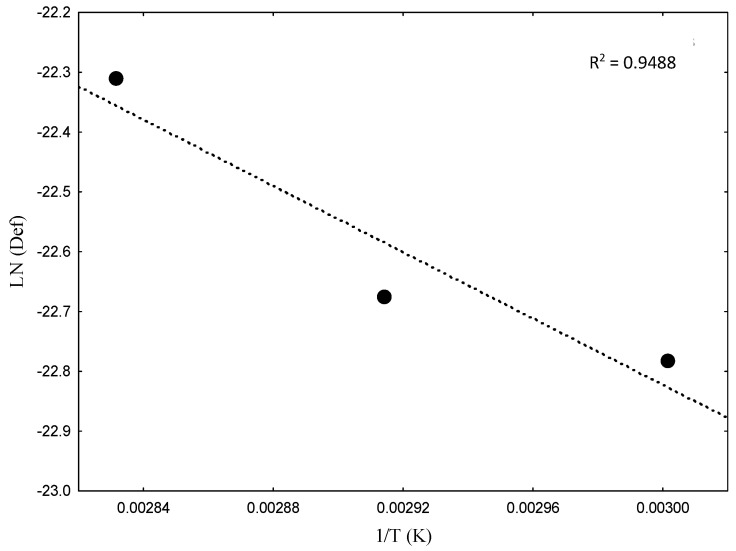
Arrhenius representation for WBP at temperature ranges of 60, 70, and 80 °C. WBP, whole beetroot paste; *R*^2^, coefficient of determination.

*D_ef_* is a measure of diffusion efficiency, considering all factors that affect water migration. It is crucial for the development and selection of appropriate equipment for the raw material, as well as for a complete understanding of its use [20,54]. The *D_ef_* values in this experiment increased with rising temperature, where 60 °C = 1.27 × 10^−9^ m^2^ s^−1^, *R*^2^ = 0.9348; 70 °C = 1.47 × 10^−9^ m^2^ s^−1^, *R*^2^ = 0.9536; 80 °C = 2.04 × 10^−9^ m^2^ s^−1^, and *R*^2^ = 0.9317. Therefore, it can be observed that the results were higher than the general range of 10^−11^ to 10^−9^ m^2^ min^−1^ for agricultural products [55], which may be associated with the occurrence of more efficient water diffusion channels due to increased matrix porosity [56]. Figure 2 shows *ln*(*D_ef_*) as a function of the inverse of the absolute temperature (T).

There was a reduction in *ln(D_ef_)* as the inverse of the temperature increased. The result varied between −22.78 for 60 °C and −22.30 for 80 °C. The E_a_ of WBP was 29.34 KJ mol^−1^, like the results of other plant matrices, such as olive pomace—29.06 KJ mol^−1^ [57], Brazilian *Lippia alba* leaves—31.79 KJ mol^−1^ [58], and apple slices—13.04–33.52 KJ mol^−1^ [59]. This energy measure allows for predicting and controlling the process speed, and no other studies measuring the E_a_ of WBP were found, making this result novel.

### 3.3. Quality Characterization of Whole Beetroot Paste (WBP) and Food Flour

The quality analyses of WBP and food flour are presented in Table 2.

The water content of raw beetroot (86%; [60]) was preserved in the WBP, despite the peel and the application of the appropriate technological process. After drying, the food flour showed a reduction in moisture content (3.17%) and, consequently, a substantial increase in ash content (6.15%). Silva et al. [9]) reported a higher moisture content (8.42%) than the present study when analyzing beetroot flour subjected to a drying temperature of 70 °C. Pasa et al. [61] obtained a similar range of ash values (5.5 to 7.1%) to the present study when evaluating beetroot flour obtained at different drying times at 75 °C.

The convective drying process directly influenced the TTA and a_w_ values, suggesting better stability against the metabolic activity of microorganisms and chemical or biochemical reactions for the dried product. The pH values for WBP and food flour were closer to neutrality compared to the results found by Farias et al. [10] when they analyzed beetroot peel flour (5.98). The variation in quality parameters is related to factors such as soil type, cultivation, planting season, planting location, and marketing conditions [28]. A pH between 5.0 and 6.0 also favors the maintenance of the color of pigments [62,63] such as betalain, present in beetroot.

The physical properties indicated results like those of Yashiki and Triboli [53] in ρ*B* (0.46) and ρ*C* (0.67), who evaluated yam flour obtained by lyophilization. The CIn and HR values were classified as excellent by the United States Pharmacopeia [64], which recommends values ≤ 10 as parameters for the flow and fluidity of a particulate material. They were also classified as having low cohesiveness by Santhalakshmy et al. [65], who recommend values below 1.2 in this category, with particle size and shape being determinants [66].

The food flour showed a good adsorption capacity, high S and W in water, and low I. Lannes and Medeiros [40] reported that powders with a good W take up to 5 min for 90% of the sample (powder) to be immersed in water. Similar S values were found by Franco et al. [67] when evaluating powdered yacon obtained by foam layer drying (80.89 to 84.16%). Feitosa et al. [68] also obtained low I (32.27 to 37.78%) for myrtle powders (*Eugenia gracillima* Kiaersk.).

### 3.4. Flour Stability in Laminated and Plastic Flexible Packaging

**Figure 3:**
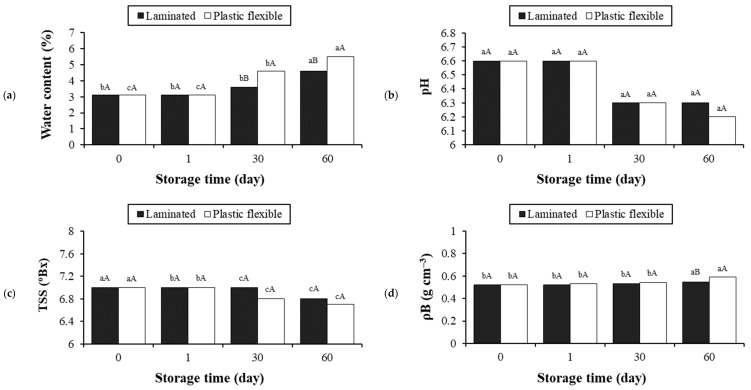

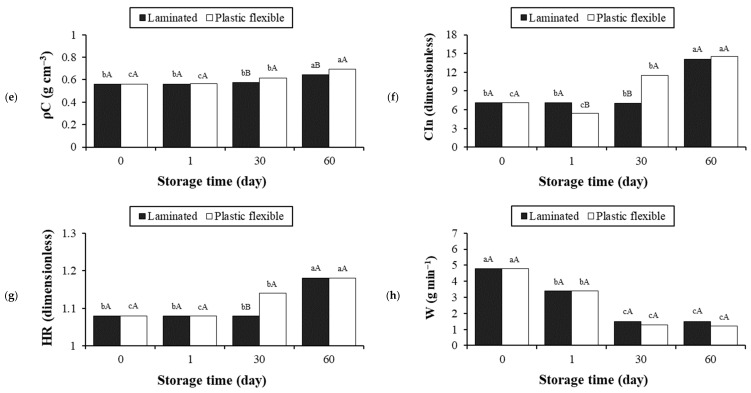
Results of water content (**a**), pH (**b**), TSS (**c**), ρB (**d**), ρC (**e**), CIn (**f**), HR (**g**), and W (**h**) for the stability of flours in laminated and plastic flexible packaging during 60 days of storage (average of 25 ± 2 °C). TSSs, total soluble solids; ρB, bulk density; ρC, tapped density; CIn, Carr index; HR, Hausner ratio; W, wettability. a–c: different lowercase letters at different storage times denote difference (*p* < 0.05; Tukey’s test); A,B: different capital letters at the same storage time denote difference (*p* < 0.05; Tukey’s test).

Figure 3 shows the results for water content (a), pH (b), TSS (c), ρ*B* (d), ρ*C* (e), CIn (f), HR (g), and W (h) for the stability of flours in laminated and plastic flexible packaging. The different packaging at room temperature (average of 25 ± 2 °C) had a significant influence (*p* < 0.05) at all storage times on most of the parameters.

The selected drying process was effective, resulting in a 96.5% reduction in the water content of the paste compared to the beetroot flour (Table 2). During the storage period, the increase in moisture content was significantly higher (*p* < 0.05) in the plastic flexible packaging. This directly interferes with stickiness, clumping, pathogen multiplication, and the quality of the product’s shelf life, although the low water content is an indication of the amorphous state [69,70,71]. Consequently, the TSS levels of the flours experienced a substantial reduction after 60 days of storage, with no statistically significant difference between the packaging types (*p* > 0.05).

Significant differences between the packaging types (*p* < 0.05) were observed only in the ρB and ρC parameters after 60 days of flour storage. Conversely, the pH and W parameters were not significantly affected during the storage of beetroot flour in different types of packaging (*p* > 0.05), with the former remaining close to neutrality (pH = 7, Table 2). The CIn values were classified as excellent throughout the storage period, according to the variation between 5 and 15% established by Villanova et al. [72]. Again, smaller fluctuations in physicochemical parameters were observed for the laminated packaging in HR. According to Santhalakshmy et al. [65], powders with an HR < 1.2 are classified as having low cohesiveness, an HR between 1.2 and 1.4 as having intermediate cohesiveness, and an HR > 1.4 as having high cohesiveness.

## 4. Conclusions

The Page model can be considered the best empirical model for predicting the drying kinetics of whole red beetroot paste at 60 °C. During storage, the laminated packaging proved to be more effective in controlling the physicochemical parameters of whole red beetroot flour, especially regarding the control of hygroscopicity. This beetroot product could serve as an alternative to traditional flours, with the advantage of being gluten-free and free of animal products. Future research is recommended to explore the sensory properties and applications of beetroot powders in biodegradable films.

## Figures and Tables

**Table 1:** Fit of empirical mathematical models for the drying of WBP.

Model	T (°C)	Constants						*R* ^2^	MSD	*X*^2^ (×10^−4^)
		*a*	*b*	*k*	*k*_1_	*n*	*c*			
Page	60	-	-	0.000230	-	1.494825	-	0.99864	0.0194	4.0333
	70	-	-	0.001131	-	1.281114	-	0.99322	0.0396	17.0401
	80	-	-	0.000346	-	1.598761	-	0.99816	0.0227	5.6476
Midilli	60	0.735224	−0.000894	−0.175843	-	0.000001	-	0.85066	0.1952	44.3504
	70	0.738498	−0.001995	−0.183674	-	0.000000	-	0.95599	0.0833	11.3400
	80	0.980643	−0.000052	0.000025	-	1.648398	-	0.99869	0.0160	4.2172
Lewis	60	-	-	0.003471	-	-	-	0.98273	0.0687	48.9230
	70	-	-	0.004884	-	-	-	0.98596	0.0569	33.6900
	80	-	-	0.006508	-	-	-	0.97654	0.0805	67.8266
Two terms	60	0.524049	0.545877	0.003830	0.003830	-	-	0.98784	0.0577	38.8660
	70	0.520622	0.496003	0.005001	0.005001	-	-	0.98628	0.0562	35.9094
	80	0.539035	0.539035	0.007094	0.007098	-	-	0.98410	0.0664	50.7679
Logarithm	60	1.219157	−0.169168	0.002856	-	-	-	0.99352	0.0422	19.3030
	70	1.262024	−0.274728	0.003157	-	-	-	0.99521	0.0333	12.6088
	80	1.272281	−0.205391	0.005188	-	-	-	0.99193	0.0474	25.8775

WBP, whole beet paste; *a*, *b*, *k*, *k*_1_, *n*, and *c*, equation constants; *R*^2^, determination coefficient; MSD, Mean Square Deviation; *X*^2^, chi-square; -, not estimated.

**Table 2:** Physicochemical and physical properties of the paste and food flour.

Parameters	Whole Beet	
	Paste	Food Flour
*Physicochemical properties*		
Water content (%)	87.40 ± 0.10	3.17 ± 0.20
Ash (%)	0.17 ± 0.02	6.15 ± 0.28
pH	6.50 ± 0.00	6.47 ± 0.06
TTA (% Citric acid)	0.37 ± 0.00	0.61 ± 0.00
TSS (°Brix)	10.00 ± 0.00	6.69 ± 0.00
a_w_	0.638 ± 0.030	-
*Physical properties*		
ρ*B* (g cm^−3^)	-	0.52 ± 0.02
ρ*C* (g cm^−3^)	-	0.56 ± 0.03
CIn	-	8.60 ± 0.28
HR	-	1.09 ± 0.00
W (g min^−1^)	-	4.75 ± 0.47
S (%)	-	90.33 ± 0.22
I (%)	-	32.62 ± 0.05

TTA, total titratable acidity; TSS, total soluble solids; a_w_, water activity; ρB bulk density; ρC compressed density; CIn, Carr index; HR, Hausner ratio; W, wettability; S, solubility; I, insolubility; -, not estimated.

## Data Availability

The data that support the findings of this study are available from the corresponding author (Bruno Fonsêca Feitosa, brunofonsecafeitosa@live.com) upon reasonable request.
